# A resource of large-scale molecular markers for monitoring *Agropyron cristatum* chromatin introgression in wheat background based on transcriptome sequences

**DOI:** 10.1038/s41598-017-12219-4

**Published:** 2017-09-20

**Authors:** Jinpeng Zhang, Weihua Liu, Yuqing Lu, Qunxing Liu, Xinming Yang, Xiuquan Li, Lihui Li

**Affiliations:** Institute of Crop Science, Chinese Academy of Agricultural Sciences, Beijing, 100081 China

## Abstract

*Agropyron cristatum* is a wild grass of the tribe Triticeae and serves as a gene donor for wheat improvement. However, very few markers can be used to monitor *A. cristatum* chromatin introgressions in wheat. Here, we reported a resource of large-scale molecular markers for tracking alien introgressions in wheat based on transcriptome sequences. By aligning *A. cristatum* unigenes with the Chinese Spring reference genome sequences, we designed 9602 *A. cristatum* expressed sequence tag-sequence-tagged site (EST-STS) markers for PCR amplification and experimental screening. As a result, 6063 polymorphic EST-STS markers were specific for the *A. cristatum* P genome in the single-receipt wheat background. A total of 4956 randomly selected polymorphic EST-STS markers were further tested in eight wheat variety backgrounds, and 3070 markers displaying stable and polymorphic amplification were validated. These markers covered more than 98% of the *A. cristatum* genome, and the marker distribution density was approximately 1.28 cM. An application case of all EST-STS markers was validated on the *A. cristatum* 6 P chromosome. These markers were successfully applied in the tracking of alien *A. cristatum* chromatin. Altogether, this study provided a universal method of large-scale molecular marker development to monitor wild relative chromatin in wheat.

## Introduction

Wild relatives of crops contain broad genetic diversity and serve as gene donors to crops^1^. In the tribe Triticeae, *Agropyron cristatum* is a wild relative of wheat and an elite gene donor for wheat improvement. In addition to its resistance to drought and cold and its moderate tolerance to salinity^2–4^, *A. cristatum* carries many useful traits, including resistance to barley yellow dwarf, wheat streak mosaic virus^5^, leaf rust^6^, and powdery mildew^7^ as well as large spike phenotype^8^, making it a powerful genetic reservoir for the improvement of wheat^6,9^.

Due to the abundance of diversified genetic resources of *A. cristatum*, wide crosses between common wheat and *A. cristatum* have been conducted to transfer desirable traits into common wheat^6,8,10–16^. Introducing *A. cristatum* alien chromosomes into common wheat depends on cytological genetic technology and molecular markers to track the alien chromosome fragment. In general, C-banding and genomic *in situ* hybridization (GISH) analyses are powerful cytological techniques for the detection of alien chromatin in wheat backgrounds^17^. However, these classic detection methods rely on complex experimental procedures and the use of fluorescence microscopes. Conversely, molecular markers can be detected rapidly and operated easily using only thermal cyclers to produce high throughput. Until now, due to the lack of genomic information, only limited molecular markers exist for *A. cristatum*. Only a few sequence-tagged site (STS) markers, sequence characterized amplified region (SCAR) markers from repeat sequences, random amplified polymorphic DNA (RAPD) markers and amplified fragment length polymorphism (AFLP) markers have been reported in *A. cristatum* ^18–20^. Chen *et al*. (1994) used a set of assigned wheat random fragment length polymorphism (RFLP) probes for detecting and establishing the homoeology of alien *A. cristatum* chromosomes^19^. Wu *et al*. (2010) reported three SCAR markers specific to the P genome repeat sequences of *A. cristatum* originating from a RAPD assay^18^. Luan *et al*. (2010) reported several 6P chromosome-specific STS markers using the suppression subtractive hybridization method^16^. Han *et al*. (2015) used the degenerate oligonucleotide-primed (DOP)-PCR products of microdissected 6PS as a fluorescence *in situ* hybridization (FISH) probe to identify P genome chromatin in wheat-*A. cristatum* introgression lines, but the method relied on cytological techniques^21^.

The large quantity of translocation lines carrying alien P chromatin of *A. cristatum* have been produced by irradiation or *Aegilops* gametocidal chromosome methods in our lab^14–16^. Hence, the heavy abundance of identification tasks requires the development of large amounts of molecular markers to efficiently track *A. cristatum* alien chromosome fragments in a wheat background. The goal of this study was to develop large-scale molecular markers for tracking *A. cristatum* alien introgressions in a wheat background based on transcriptome sequences. This study provides a comprehensive molecular marker resource and presents a universal application case of molecular marker development in wide crosses based on transcriptome sequencing technology.

## Results

### Sequence alignment analysis and primer design

**Figure 1:**
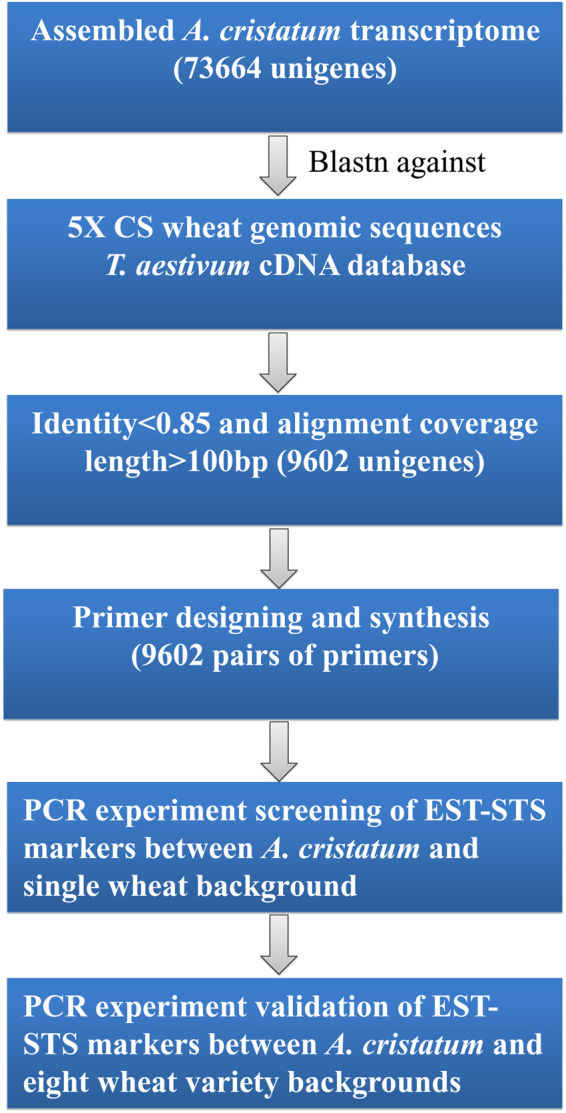
The workflow of the EST-STS marker development.

We previously reported that more than 90 million transcriptome reads from the flag leaf and young spike tissues of the representative tetraploid *A. cristatum* accession No. Z559 were assembled into 73664 unigenes^22^. Considering a 93.8% average identity between *A. cristatum* unigenes and wheat transcripts, unigenes with high identity were removed to increase the polymorphic rate of primer screening. Hence, we first conducted the alignment analysis using the Basic Local Alignment Search Tool for nucleotides (BLASTn) on the 73664 unigenes with the *Triticum aestivum* cDNA database and 5X CS wheat genomic DNA sequences^23^, after which the remaining unigenes that had less than 85% identity were used for primer design according to the outline shown in Fig. 1. Finally, we selected 9602 unigenes that had low identity for primer design. All primer names and sequences are provided in Supplementary Table 1. Most primers were designed by the high-throughput web application platform BatchPrimer3, using the generic primer method and the default parameters. A few primers were designed manually that exhibited insertion-deletion (InDel) variation between *A. cristatum* unigenes and wheat transcripts. The workflow of the expressed sequence tag-STS (EST-STS) marker development is shown in Fig. 1.

### PCR experiment screening of EST-STS markers specific for the P genome in the single-receipt wheat background

We selected *A. cristatum* Z559 as a positive control and the common wheat parent Fukuho (from the original wide cross) as a negative control for the experimental screening of specific primers of the P genome in the wheat background. First, all 9602 primer pairs were amplified in two control DNA templates, and polymorphic PCR product bands were distinguished on non-denaturing polyacrylamide gels. Two types of markers were screened: dominant and co-dominant markers. The dominant markers amplify only within the *A. cristatum* template, whereas co-dominant markers amplify polymorphic products in both the *A. cristatum* and wheat templates. As a result, 6063 polymorphic markers were specific for the *A. cristatum* genome (Supplementary Table 2). The screening rate reached 64.5%. More than 30% of the markers were functionally annotated using the NCBI nonredundant (nr) protein database, but the other 70% had no homologous genes in any database. Two thousand ninety-five markers without annotation belonged to predicted long intergenic noncoding RNAs (lincRNAs) (Supplementary Table 2). We previously showed that these lincRNAs are specific to *A. cristatum* as opposed to other Triticeae species, including wheat, rye and barley^22^. Hence, these 2095 markers will be useful for identifying the *A. cristatum* P genome among diversified Triticeae grasses.

To evaluate the coverage rate and probable distribution density of these markers on *A. cristatum* chromosomes, a virtual chromosome location map was constructed by anchoring these markers onto the wheat population sequencing (POPSEQ) genetic map^24^. A total of 1951 EST-STS markers were successfully anchored onto the wheat POPSEQ genetic map (Supplementary Table 3). These markers are covered more than 98% of the *A. cristatum* genome, and the marker distribution density was 1.28 cM (Table 1). The virtual distribution map suggests these markers have high density coverage on *A. cristatum* chromosomes. Using the wheat POPSEQ genetic map, we isolated 584 EST-STS markers as conserved loci between the P genome and the A, B, and D genomes, which will be useful for the homologous identification of *A. cristatum* alien chromosomes of different addition lines.

**Table 1:** Virtual chromosome location of EST-STS markers of *A. cristatum* in the reference wheat genome

Homologous groups	Chromosome	Number of conserved markers	Marker coverage rate	Marker density (cM/marker)
1	1A(106),1B(123),1D(105)	74	100%	1.43
2	2A(146),2B(178),2D(140)	104	98%	0.83
3	3A(69),3B(158),3D(99)	53	98%	1.88
4	4A(92),4B(111),4D(59)	42	96%	1.29
5	5A(77),5B(143),5D(146)	63	99%	1.48
6	6A(240),6B(191),6D(263)	177	99%	0.64
7	7A(118),7B(110),7D(195)	71	97%	1.40

The number within parentheses denotes the number of markers located on the corresponding wheat chromosome.

### Validation of EST-STS markers specific for the P genome in multiple wheat backgrounds

To validate the specificity of EST-STS markers for the P genome of *A. cristatum* in wheat genetic backgrounds and remove false-positive markers, we collected eight domestic and international common wheat varieties, including Fukuho, Chinese Spring (CS), Gaocheng 8901, Xiaoyan 6, Hi-Line, CMH83.605, McGuire, and Lovrin 10, for the further experimental validation. The wheat varieties that have high genetic variation served as negative controls to eliminate the false-positive disturbance from the wheat background. Accounting for 1B/1R translocations distributed widely throughout commercial varieties^25,26^, we also increased the representative variety Lovrin 10 that has the 1B/1R translocation for detection. A total of 4956 markers with polymorphism between *A. cristatum* and Fukuho were randomly selected for validation. As a result, 3070 EST-STS markers that exhibited stable amplification and clear PCR product bands were screened. The screening rate reached 61.9%. The highly stringent markers are provided in Supplementary Table 4. Based on these screening results, twenty-one markers were amplified in both *A. cristatum* and the Lovrin 10 1B/1R translocation line. These EST-STS markers included AgC5106, AgC12260, AgC14448, AgC23390, AgC23424, AgC24347, AgC24610, AgC24754, AgC24816, AgC25726, AgC26732, AgC30508, AgC31937, AgC36971, AgC49315, AgC50266, AgC50467, AgC52107, AgC53511, AgC54696 and AgC71190, which could be detected in both 1B/1R translocation and *A. cristatum*. We suggested that the 21 EST-STS markers should be ruled out in the detection of wheat backgrounds with the 1B/1R translocation.

### EST-STS marker screening specific for *A. cristatum* chromosomes from addition lines

**Figure 2:**
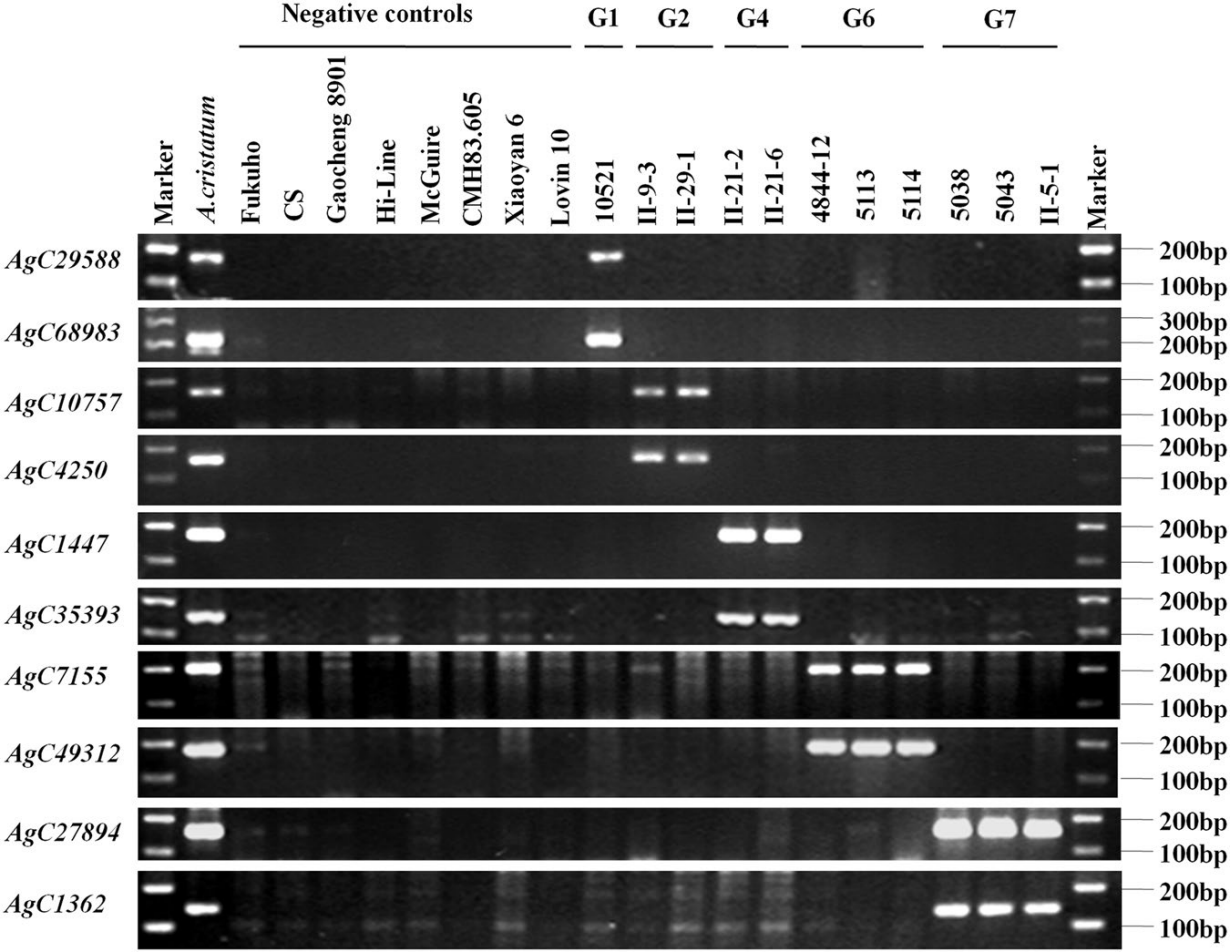
The screening of EST-STS markers specific for *A. cristatum* addition lines in a wheat genetic background. The amplification product of EST-STS markers were run on 2% agarose gel. G1, G2, G4, G6, and G7 indicate the homologous groups of alien chromosomes of *A. cristatum*. The full-length gels are included in the Supplementary Information file.

To screen the EST-STS markers specific for single chromosomes of *A. cristatum* in the wheat genetic background, we collected from our lab 11 addition lines, including 5 homologous groups, for screening the specific marker for P chromosomes (Table 2). Two markers were screened for each chromosome, including chromosomes 1P, 2P, 4P, 6P and 7P, from the addition lines. These markers were highly amplified in both *A. cristatum* and the addition lines, whereas no amplification was observed in the eight wheat varieties that have broad genetic diversity. Among these markers, AgC29588 and AgC68083 were specific for chromosome 1P, AgC10757 and AgC4250 were specific for chromosome 2P, AgC1447 and AgC35393 were specific for chromosome 4P, AgC7155 and AgC49312 were specific for chromosome 6P, and AgC27894 and AgC1362 were specific for chromosome 7P (Fig. 2). These markers will be very useful for distinguishing the specific chromosomes in wide crosses of common wheat and *A. cristatum*.

**Table 2:** Plant materials used in this study

Material name	Chromosome composition	Type of material	Homologous group of wild relative	Origin country
Z559	2n = 4X = 28P	*A. cristatum*		China
10521	2n = 6X = 42W + 2P	addition line	1P	China
II-9-3	2n = 6X = 42W + 2P	addition line	2P	China
II-29-1	2n = 6X = 42W + 2P	addition line	2P	China
II-21-2	2n = 6X = 42W + 2P	addition line	4P	China
II-21-6	2n = 6X = 42W + 2P	addition line	4P	China
4844-12	2n = 6X = 42W + 2P	addition line	6P	China
5113	2n = 6X = 42W + 2P	addition line	6P	China
5114	2n = 6X = 42W + 2P	addition line	6P	China
5038	2n = 6X = 42W + 2P	addition line	7P	China
5043	2n = 6X = 42W + 2P	addition line	7P	China
II-5-1	2n = 6X = 42W + 2P	addition line	7P	China
Fukuho	2n = 6X = 42W	wheat landrace		Japan
Chinese Spring	2n = 6X = 42W	wheat landrace		China
Gaocheng 8901	2n = 6X = 42W	wheat landrace		China
Xiaoyan 6	2n = 6X = 42W	commercial wheat variety		China
Hi-Line	2n = 6X = 42W	commercial wheat variety		USA
CMH83.605	2n = 6X = 42W	commercial wheat variety		CIMMYT
McGuire	2n = 6X = 42W	commercial wheat variety		USA
Lovrin 10	2n = 6X = 42W	1BL.1RS translocation		Romania

### EST-STS marker validation in *A. cristatum* chromosome 6P

**Figure 3:**
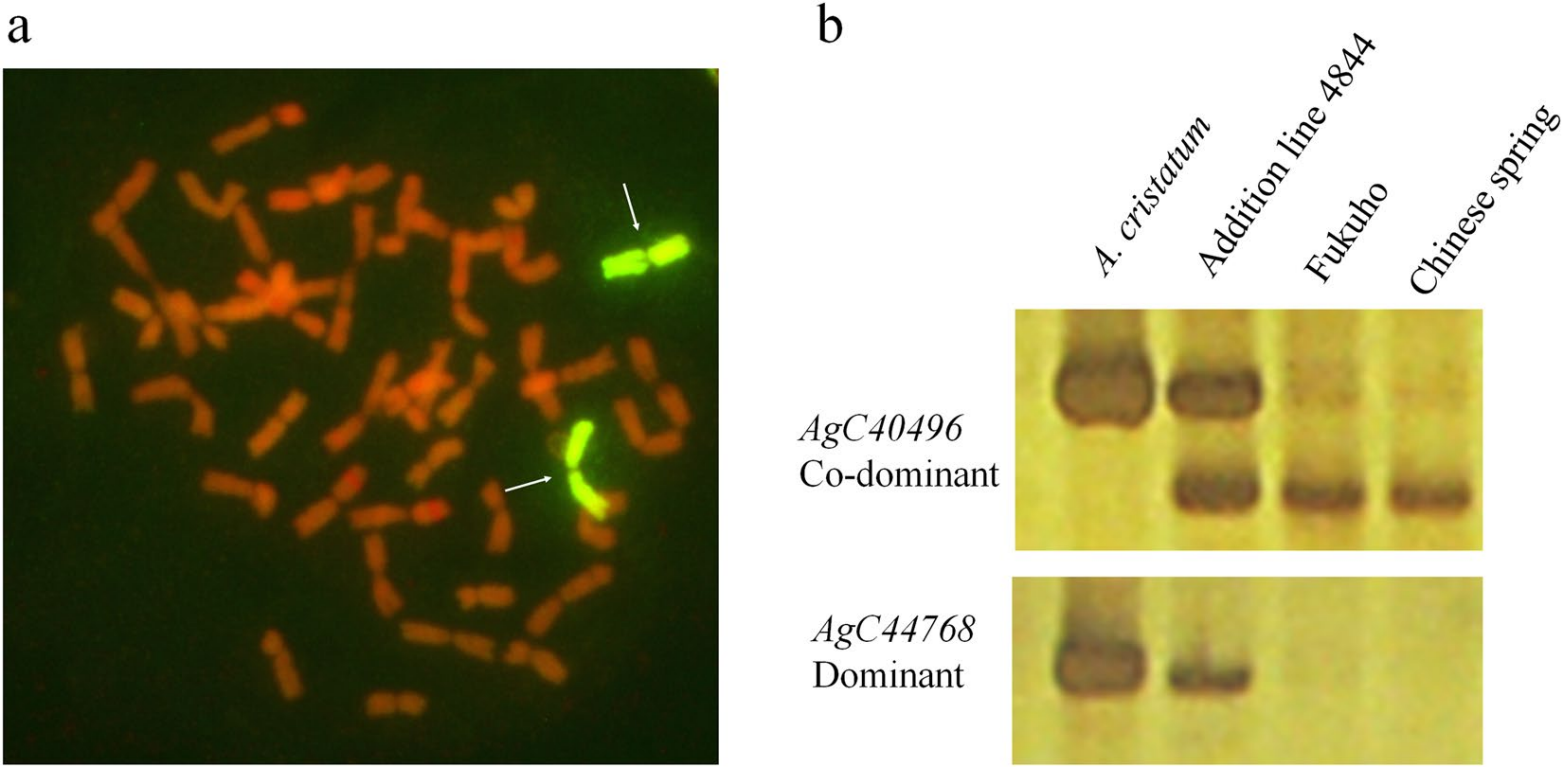
*Agropyron cristatum* chromosome 6P-specific EST-STS marker screening in a wheat background. (**a**) GISH detection showed 21 pairs of wheat chromosomes and one pair of 6P chromosomes in addition line 4844-12. The arrowheads indicate the addition of chromosome 6P by the green fluorescent signal. (**b**) The amplification product of EST-STS markers was run on 8% non-denaturing polyacrylamide gel. *Agropyron cristatum* and addition line 4844-12 are positive controls, and wheat varieties Fukuho and Chinese Spring are negative controls.

To validate the screening rate and efficiency of EST-STS markers in wheat backgrounds, we selected chromosome 6P, which carries a high number of grain-related traits, as a validation case^8^. We screened all EST-STS markers on chromosome 6P of addition line 4844-12. All 9602 markers were screened among the four DNA samples, including *A. cristatum*, 6P addition line 4844-12, Fukuho (the receiving parent of the wide cross) and Chinese Spring. GISH detection first proved addition line 4844-12 contained a pair of *A. cristatum* chromosome 6P (Fig. 3a). The experimental screening was then performed for all 9602 pairs of primers. PCR amplification band types of EST-STS markers were divided into co-dominant and dominant types (Fig. 3b). For example, the co-dominant marker AgC40496 amplified two different bands from *A. cristatum* and common wheat, whereas the dominant marker AgC44768 only amplified one band in *A. cristatum* and none in wheat (Fig. 3b). Finally, after high-stringency experiment screening, 680 EST-STS markers specific for chromosome 6P were screened from 9602 primers, resulting in a 7.08% screening rate (Supplementary Table 5). These markers have been widely applied for monitoring chromosome 6P segments in addition lines, deletion lines and translocation lines^27–30^.

To evaluate the probable order of the 680 EST-STS markers on chromosome 6P, the homologous relationships of the 680 EST-STS markers on chromosome 6P were evaluated using BLASTn against wheat and barley genomes (Table 3). Three hundred fifty-nine and 259 EST-STS markers of *A. cristatum* chromosome 6P were anchored onto wheat and barley chromosomes, respectively. The highest percentages of anchored markers were 30.29% and 23.09% in the sixth homologous group of wheat and barley, respectively. According to the wheat POPSEQ genetic map of the A, B and D subgenomes^24^, three virtual genetic maps were also constructed (Supplementary Figure 1). Two hundred forty-one unique EST-STS markers were anchored onto chromosomes 6A, 6B and 6D, and 38 EST-STS markers were conserved among chromosomes 6P, 6A, 6B and 6D. The results indicate a good synteny relationship between chromosome 6P and chromosomes 6A, 6B or 6D. Hence, we confirmed the application value of all *A. cristatum* EST-STS markers in the detection of alien chromosomes in a wheat background.

**Table 3:** Comparative analysis of homologous relationships between *A. cristatum* and wheat and barley chromosomes based on the alignment of *A. cristatum* 6P-specific EST-STS markers^a^.

Species	Homologous groups	Number of anchored unigenes	Average identity (%)	Average coverage length (bp)	Percent of anchored markers
Wheat	1A,1B,1D	20	89.6	251	2.94%
	2A,2B,2D	18	89.6	211	2.65%
	3A,3B,3D	30	88.4	243	4.41%
	4A,4B,4D	19	87.1	260	2.79%
	5A,5B,5D	30	90.1	258	4.41%
	6A,6B,6D	206	91.4	335	30.29%
	7A,7B,7D	36	88.5	256	5.29%
	No hits	321			47.21%
Barley	1H	17	89.4	184	2.50%
	2H	15	88.6	165	2.21%
	3H	24	90.6	207	3.53%
	4H	11	89.0	216	1.62%
	5H	17	88.4	170	2.50%
	6H	157	91.3	286	23.09%
	7H	18	89.1	189	2.65%
	No hits	421			61.91%

^a^BLASTn alignments were conducted with an e-value threshold less than 1e-10 and an alignment coverage length of more than 100 bp.

## Discussion

Traditionally, the detection of alien chromosomes has mainly depended on chromosome banding and GISH techniques^17^. However, the two methods require complex instruments and expensive reagents. Molecular markers provide a rapid, reliable, easy, high-throughput method for detecting the alien chromosome segments. We previously showed that repeat sequence markers can detect *A. cristatum* alien chromosomes in a common wheat background^18,21^. However, repeat sequence markers are distributed throughout a whole chromosome and cannot distinguished specific chromosome fragment loci. EST-STS markers have been effective in similar studies involving *Haynaldia villosa*, *Secale cereale*, and *Psathyrostachys huashanica* ^31–33^. These markers successfully tracked specific chromosomes or homologous groups. In the present study, we demonstrated the successful development of large-scale EST-STS markers based on transcriptome sequences for tracking *A. cristatum* chromosome fragments in a wheat background. These markers originated from native transcriptome sequences of *A. cristatum* and showed three main characteristics: (i) They are monomorphic and can be tracked to specific chromosome segments; (ii) They have an estimated average marker coverage density of 98% of the *A. cristatum* genome; and (iii) They consist of native markers from the *A. cristatum* transcriptome. Among these EST-STS markers, 6063 can be used in the identification of wide crosses in the single-receipt Fukuho parent background, and 3070 *A. cristatum-*specific markers in eight wheat variety backgrounds can be applied to molecular marker-assisted selection of *A. cristatum* introgressions into commercial varieties. In the present study, these markers have been applied successfully for the physical mapping of 2P, 6P and 7P alien chromatin fragments in *A. cristatum*–wheat introgression lines^27,30,34,35^.

We previously showed that 4831 *A. cristatum* unigenes were predicted as lincRNAs and were *A. cristatum* specific compared with other Triticeae species^22^. In the present study, 2095 markers were predicted to be lincRNAs and accounted for 43% of all *A. cristatum*-specific lincRNAs. The results imply that lincRNAs should be the first choice for marker development and have the advantage of species specificity for monitoring wild relative chromatin in wheat backgrounds. Taken together, this study reported a universal method of large-scale molecular marker development for monitoring wild relative chromatin in the common wheat background based on transcriptome sequences.

In the study of wide crosses, identifying the homologous group of alien chromosomes from the Triticeae tribe is a major challenge. Successful identification leads to the effective selection and utilization of homoeologous recombination translocation lines between wheat and wild relatives^36^. In the present study, we used 6P addition line 4844-12 as a case for large-scale marker development of chromosome 6P specifically in a common wheat background and for homologous group identification. A total of 680 high-quality 6P-specific markers in the wheat genetic background provided a rapid detection platform for chromosome engineering applications, and these markers were used to track the alien 6P fragment of *A. cristatum* in the common wheat background. The positive marker screening rate in addition line 4844-12 was close to 1/14 (680/9602≈1/14) rather than 1/7, suggesting that the *A. cristatum* tetraploid in our study is not an autotetraploid but rather a segmental allotetraploid, which was in accordance with the results of a previous study^37^. After comparing 680 sequences with wheat draft genomes, a good synteny relationship was observed between *A. cristatum* chromosome 6P and wheat chromosomes 6A, 6B and 6D. The present study also supports the previous cytogenetic results regarding the replacement of chromosome 6D of wheat with chromosome 6P of *A. cristatum* in wheat–*A. cristatum* chromosome substitution lines^8^. The study provides good prospective applications for the homologous group identification of alien chromosomes in addition lines of other wild relatives based on transcriptome sequences.

## Methods

### Plant materials

The tetraploid *A. cristatum* accession No. Z559 was used as the donor parent for a wide cross between wheat and *A. cristatum* in our previous study^10^. The tetraploid *A. cristatum* (2n = 4X = 28 P) Z559 population was also selected for de novo transcriptome sequencing in our previous study^22^. Common wheat varieties including Fukuho, Chinese Spring, Gaocheng 8901, Xiaoyan 6, Hi-Line, CMH83.605, McGuire, and Lovrin 10 served as negative controls and wheat genetic backgrounds for screening P genome-specific markers. Among these varieties, Fukuho was the original maternal parent of the wide cross between common wheat and *A. cristatum*, Chinese Spring and Gaocheng 8901 were Chinese landraces, and Xiaoyan 6 was the commercial variety of China. Hi-Line, CMH83.605, McGuire and Lovrin 10 were introduced varieties from other parts of the world. Disomic addition lines of wheat and *A. cristatum* were selected for *A. cristatum* chromosome-specific marker screening in a wheat background. These addition lines include 10521 (homologous group 1), II-9-3 and II-29-1 (homologous group 2), II-21-2 and II-21-6 (homologous group 4), 4844-12, 5113 and 5114 (homologous group 6), and 5038, 5043 and II-5-1 (homologous group 7). Plant material information is listed in Table 2.

### Sequence analysis and primer design

The assembled 73664 unigenes of the *A. cristatum* transcriptome^22^ were used for alignment analysis by BLASTn against 5X CS wheat genome raw data sequences^23^ and the *T. aestivum* cDNA database (ftp://ftp.ensemblgenomes.org/pub/release-23/plants/fasta/). After removing high-identity sequences, we selected 9602 unigenes that had a nucleotide identity less than 85% for primer design to increase the polymorphism of nucleotide sequences. The large-scale resource of primer pairs was designed using the BatchPrimer3 website (http://probes.pw.usda.gov/cgi-bin/batchprimer3/batchprimer3.cgi) with the following parameters: product length of 100–300 bp, primer size of 18–24 bp (20 bp optimum) and melting temperature between 57 and 63 °C (60 °C optimum)^38^. A small amount of primers were designed manually that exhibited InDel variation between *A. cristatum* unigenes and wheat transcripts. Detailed primer lists are shown in Supplementary Table 1.

### DNA extraction, PCR experiments and STS marker screening

DNA was extracted from young leaf tissue at the seedling stage using cetyltrimethylammonium bromide (CTAB) extraction buffer according to published methods^39^. PCR experiments were performed in a 10-μL reaction volume containing 10X PCR buffer, 0.2 mmol/L dNTP, 2.5 mmol/L Mg^2+^, 0.25 μmol/L primer, 0.6 U of Taq DNA polymerase, and 50 ng of template DNA. The PCR cycling parameters consisted of one cycle at 94 °C for 3 min, 35 cycles at 94 °C for 1 min, the appropriate annealing temperature for 1 min (depending on the individual primer pair), and 72 °C for 1 min; and a final extension for 7 min at 72 °C. PCR products were electrophoresed on 8% non-denaturing polyacrylamide gels (Acr/Bis = 19:1) and detected by the silver staining method^40^ or on 2% agarose gels. The DNA template from *A. cristatum* Z559 and wheat–*A. cristatum* disomic addition lines served as positive controls, and the DNA template from the other eight common wheat varieties served as negative controls. The primers were screened for polymorphisms according to the size of PCR amplification products.

### Anchoring EST-STS markers on wheat chromosome map

Anchoring *A. cristatum* EST-STS markers onto the wheat chromosome map was performed by BLASTn alignment of *A. cristatum* unigene sequences against the Chinese Spring reference genome in accordance with the wheat POPSEQ genetic map of the A, B and D subgenomes^24^ and the barley H genome^41^. High-stringency alignment was considered when the statistics of an e-value threshold less than 1e-10 and an alignment coverage length greater than 100 bp occurred.

### Functional annotation of EST-STS markers

The functional annotation of *A. cristatum* EST-STS markers was performed in our previous study using the NCBI nr protein sequences, the Kyoto Encyclopedia of Genes and Genomes (KEGG) pathway database, the Pfam domain database and lincRNAs^22^.

### GISH analysis

GISH detection in addition line 4844-12 was performed in root tip cells using *A. cristatum* genomic DNA as a probe. Wheat genomic DNA from the receiving parent Fukuho was used for blocking. Root tip preparation and hybridization procedures were performed as described by Han *et al*. (2004)^42^.

## Electronic supplementary material

Supplementary information

Supplementary Table 1

Supplementary Table 2

Supplementary Table 3

Supplementary Table 4

Supplementary Table 5
