# Supplementary material for: A resource of large-scale molecular markers for monitoring Agropyron cristatum chromatin introgression in wheat background based on transcriptome sequences
Source: Sci Rep. 2017 Sep 20;7:11942. doi: 10.1038/s41598-017-12219-4 (PMC5607264; doi:10.1038/s41598-017-12219-4)
Supplement: Supplementary file 1 — Supplementary information [file 41598_2017_12219_MOESM1_ESM.pdf]

Supplementary information

A resource of large-scale molecular markers for monitoring *Agropyron cristatum* chromatin introgression in wheat background based on transcriptome sequences

Jinpeng Zhang[1], Weihua Liu[1], Yuqing Lu[1], Qunxing Liu[1], Xinming Yang[1], Xiuquan Li[1], Lihui Li[1*]

Affiliation

[1]Institute of Crop Science, Chinese Academy of Agricultural Sciences, Beijing, 100081, China

*Corresponding author: Email: lilihui@caas.cn, Fax: +86-10-62189650

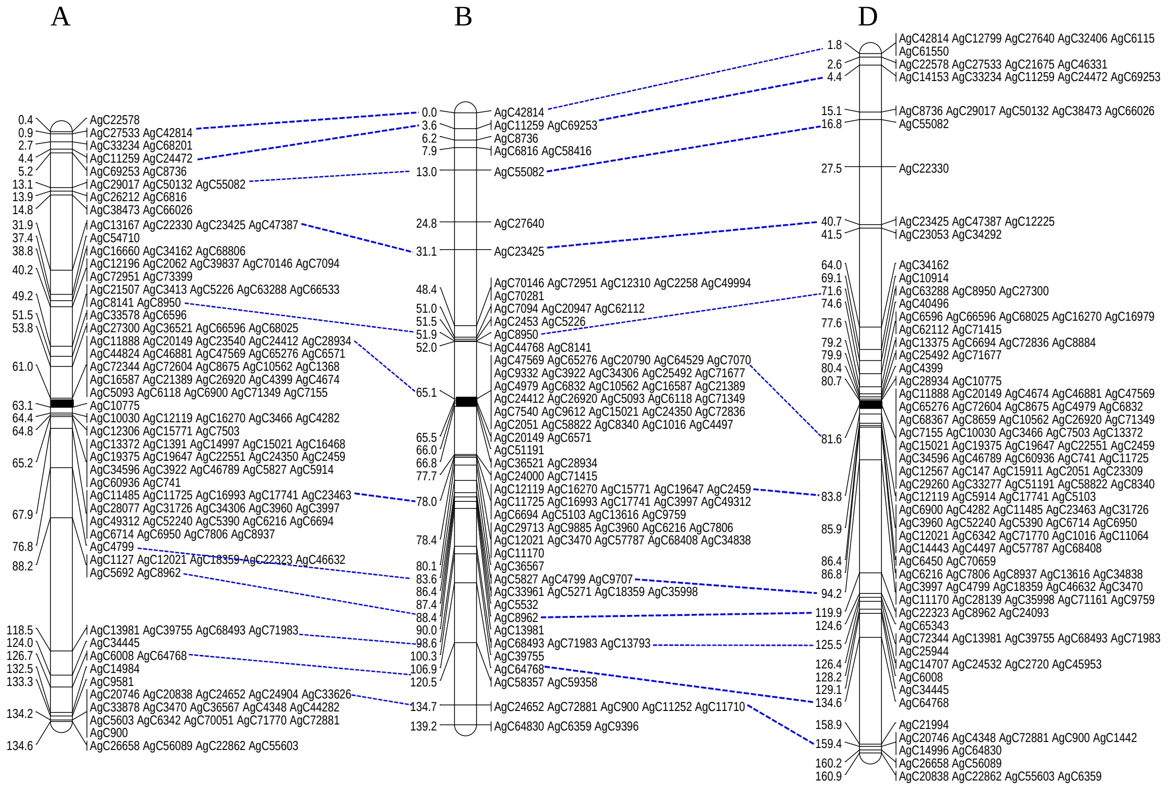

Supplementary Figure 1. A virtual chromosome location map was constructed by anchoring 680 *A. cristatum* 6P-specific EST-STS markers on 6A(A), 6B(B) and 6D(D) chromosomes of wheat POPSEQ genetic map.

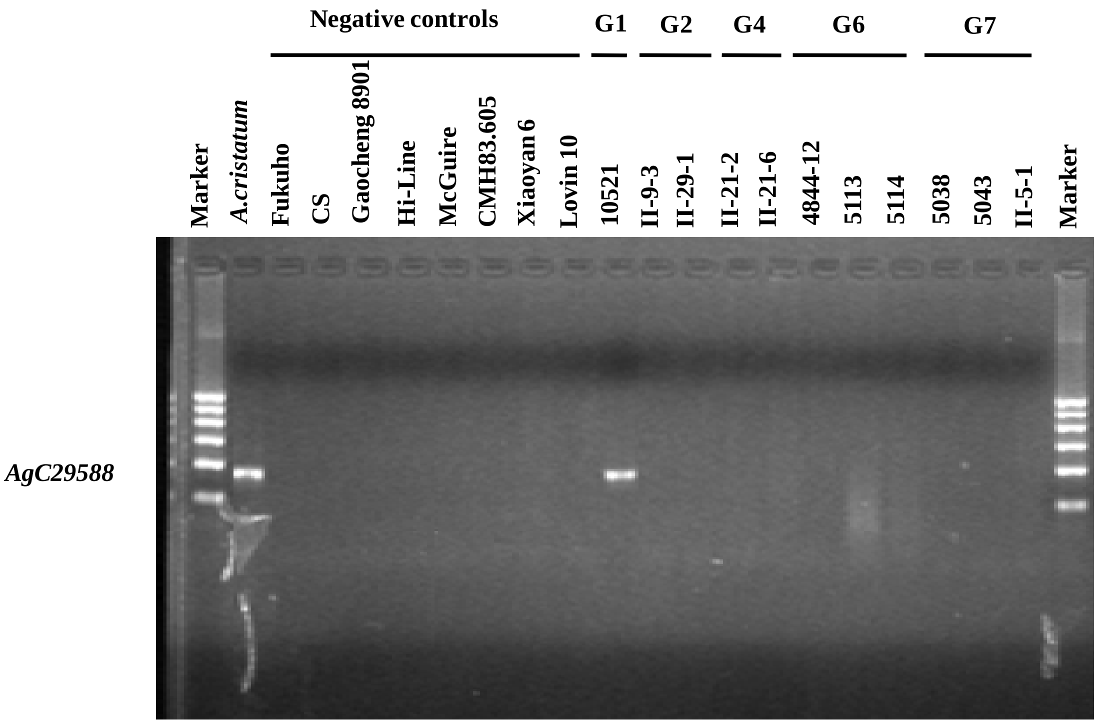

Supplementary Figure 2. The full-length gels of EST-STS markers AgC29588 specific for *A. cristatum* addition lines in wheat genetic backgrounds. The amplification product of EST-STS markers were run on 2% agarose gel. G1, G2, G4, G6, and G7 indicate the homologous groups of alien chromosomes of *A. cristatum*.

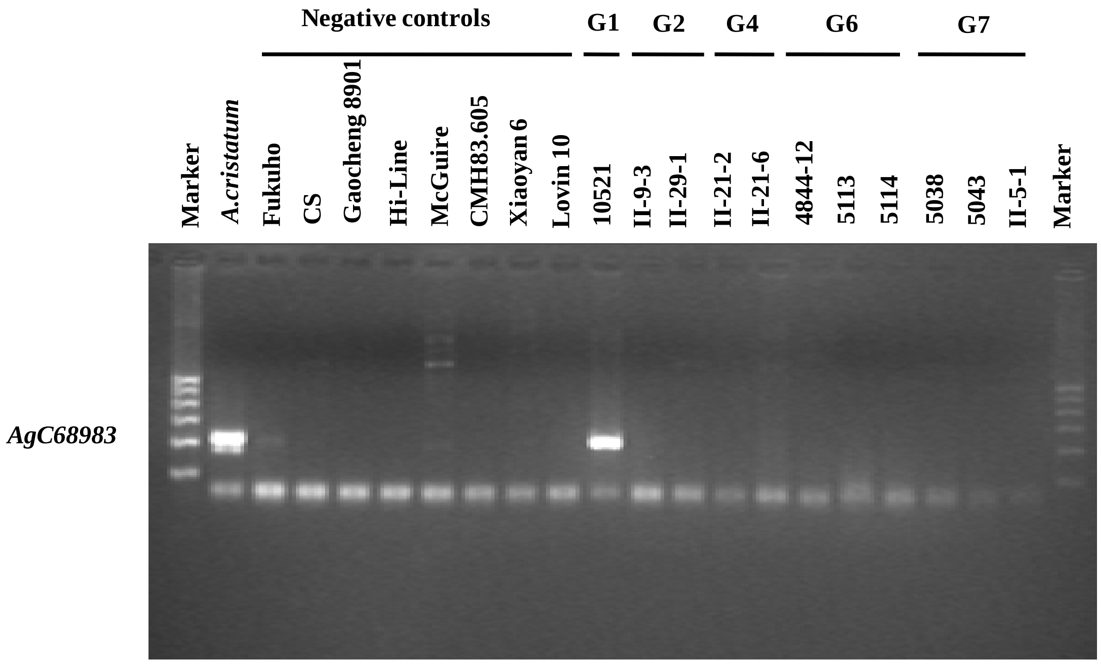

Supplementary Figure 3. The full-length gels of EST-STS markers AgC68983 specific for *A. cristatum* addition lines in wheat genetic backgrounds. The amplification product of EST-STS markers were run on 2% agarose gel. G1, G2, G4, G6, and G7 indicate the homologous groups of alien chromosomes of *A. cristatum*.

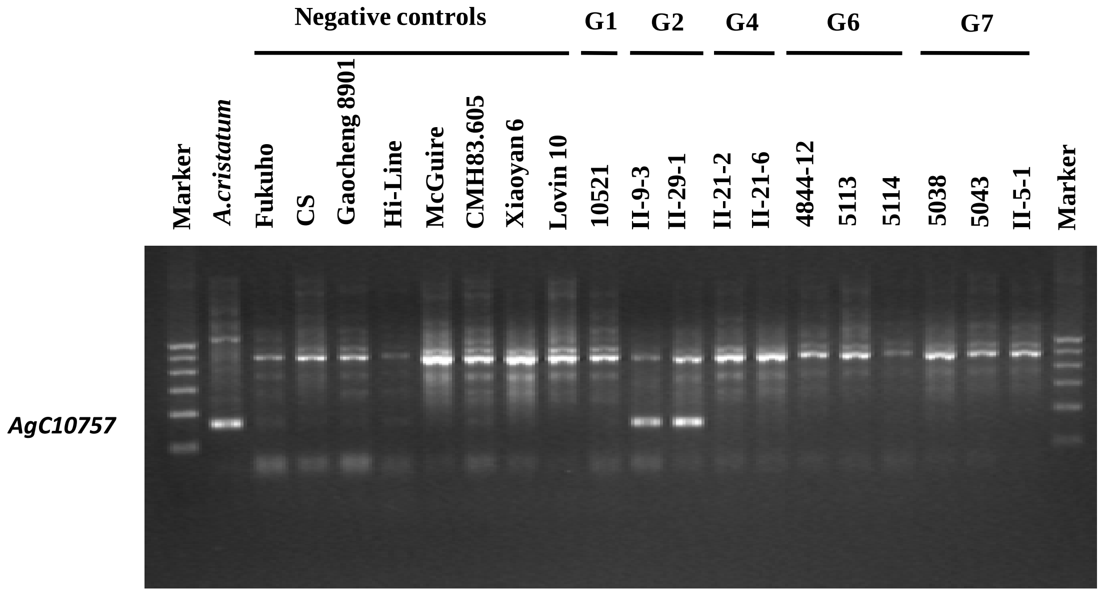

Supplementary Figure 4. The full-length gels of EST-STS markers AgC10757 specific for *A. cristatum* addition lines in wheat genetic backgrounds. The amplification product of EST-STS markers were run on 2% agarose gel. G1, G2, G4, G6, and G7 indicate the homologous groups of alien chromosomes of *A. cristatum*.

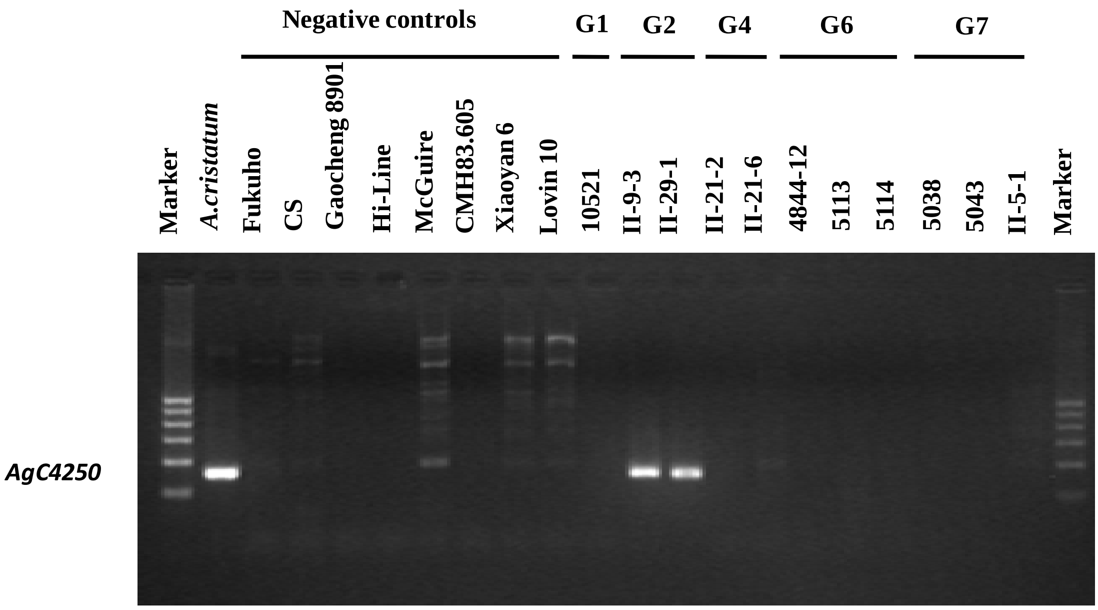

Supplementary Figure 5. The full-length gels of EST-STS markers AgC4250 specific for *A. cristatum* addition lines in wheat genetic backgrounds. The amplification product of EST-STS markers were run on 2% agarose gel. G1, G2, G4, G6, and G7 indicate the homologous groups of alien chromosomes of *A. cristatum*.

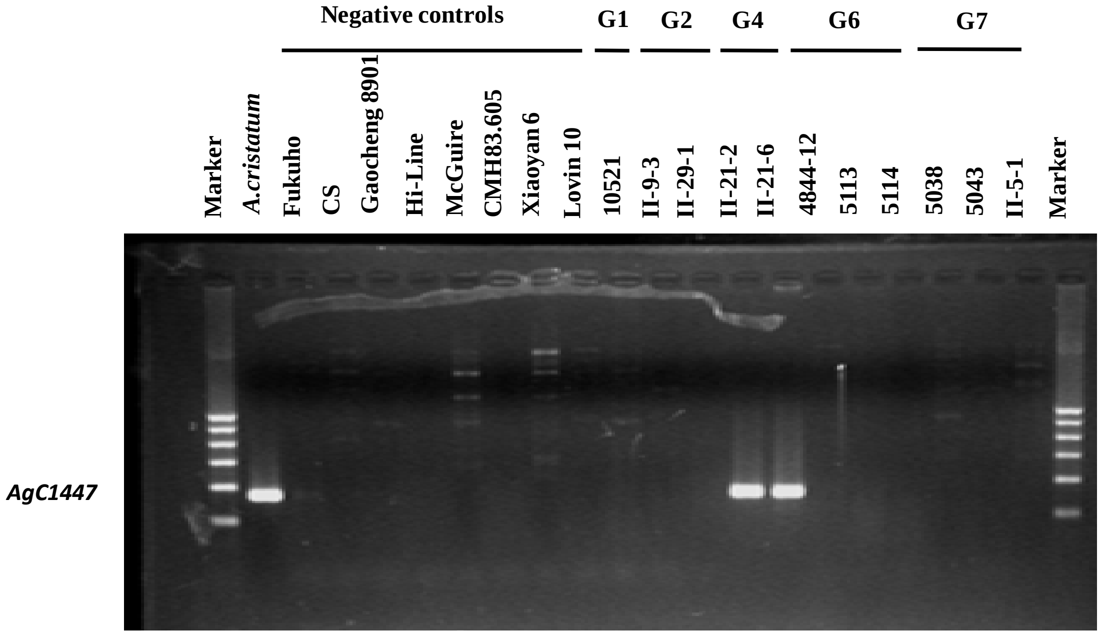

Supplementary Figure 6. The full-length gels of EST-STS markers AgC1447 specific for *A. cristatum* addition lines in wheat genetic backgrounds. The amplification product of EST-STS markers were run on 2% agarose gel. G1, G2, G4, G6, and G7 indicate the homologous groups of alien chromosomes of *A. cristatum*.

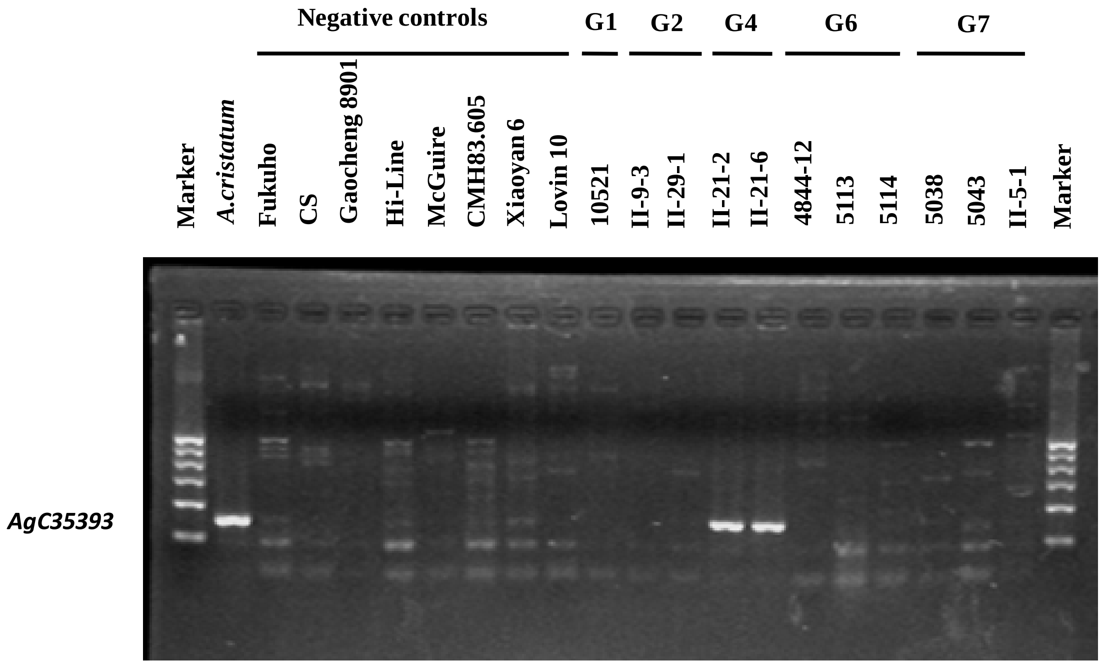

Supplementary Figure 7. The full-length gels of EST-STS markers AgC35393 specific for *A. cristatum* addition lines in wheat genetic backgrounds. The amplification product of EST-STS markers were run on 2% agarose gel. G1, G2, G4, G6, and G7 indicate the homologous groups of alien chromosomes of *A. cristatum*.

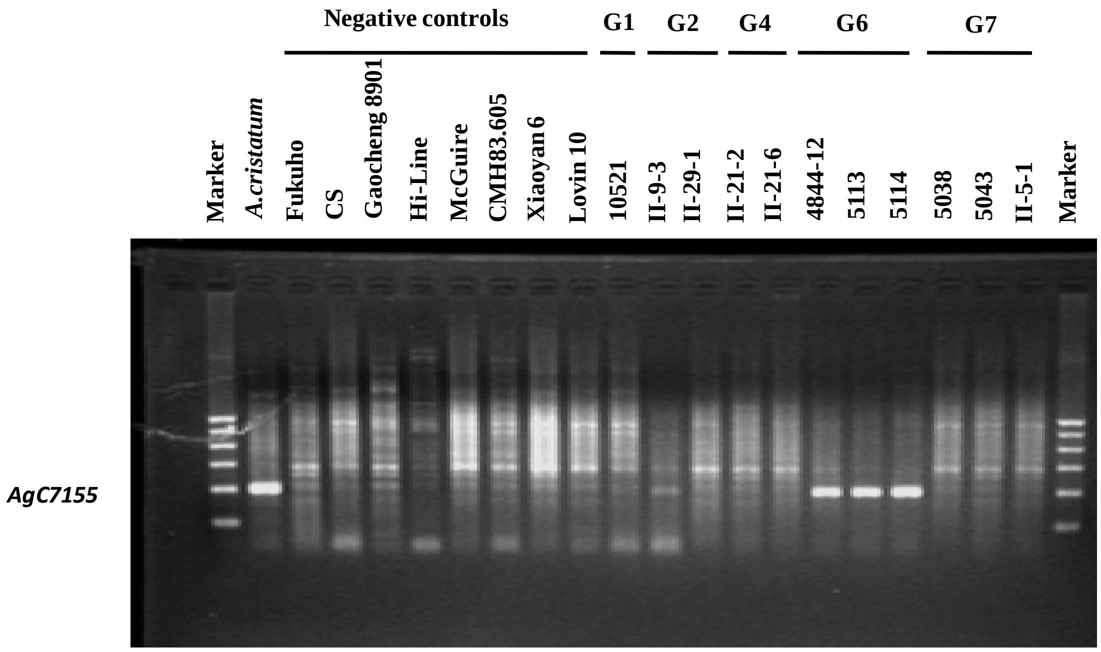

Supplementary Figure 8. The full-length gels of EST-STS markers AgC7155 specific for *A. cristatum* addition lines in wheat genetic backgrounds. The amplification product of EST-STS markers were run on 2% agarose gel. G1, G2, G4, G6, and G7 indicate the homologous groups of alien chromosomes of *A. cristatum*.

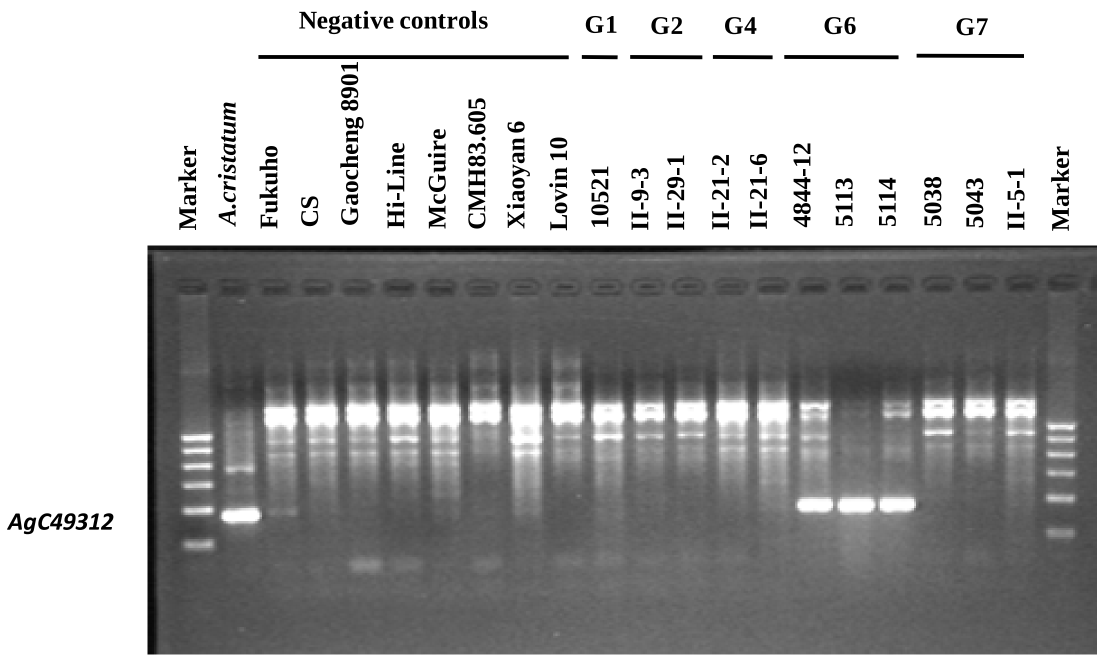

Supplementary Figure 9. The full-length gels of EST-STS markers AgC49312 specific for *A. cristatum* addition lines in wheat genetic backgrounds. The amplification product of EST-STS markers were run on 2% agarose gel. G1, G2, G4, G6, and G7 indicate the homologous groups of alien chromosomes of *A. cristatum*.

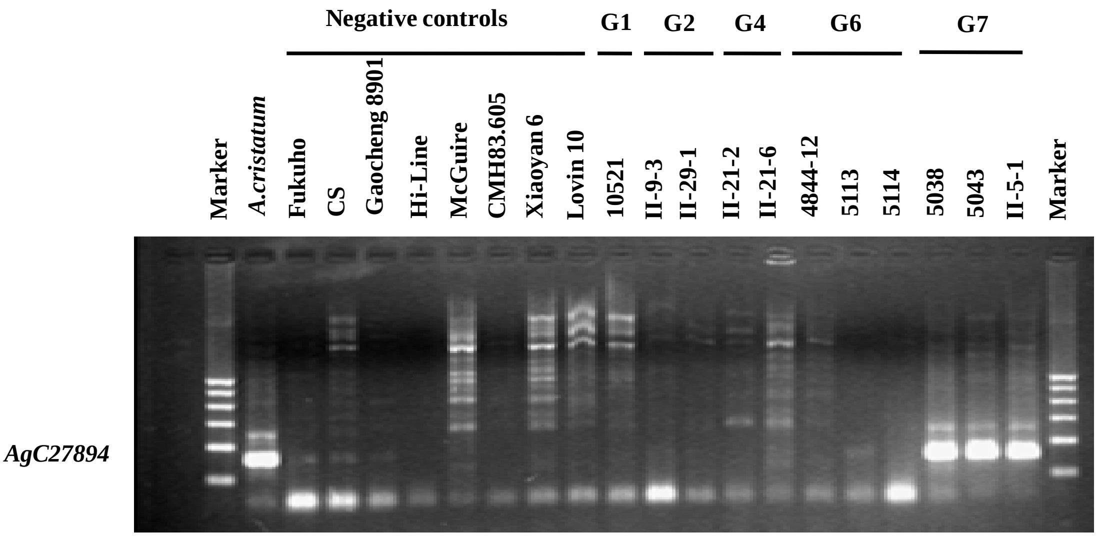

Supplementary Figure 10. The full-length gels of EST-STS markers AgC27894 specific for *A. cristatum* addition lines in wheat genetic backgrounds. The amplification product of EST-STS markers were run on 2% agarose gel. G1, G2, G4, G6, and G7 indicate the homologous groups of alien chromosomes of *A. cristatum*.

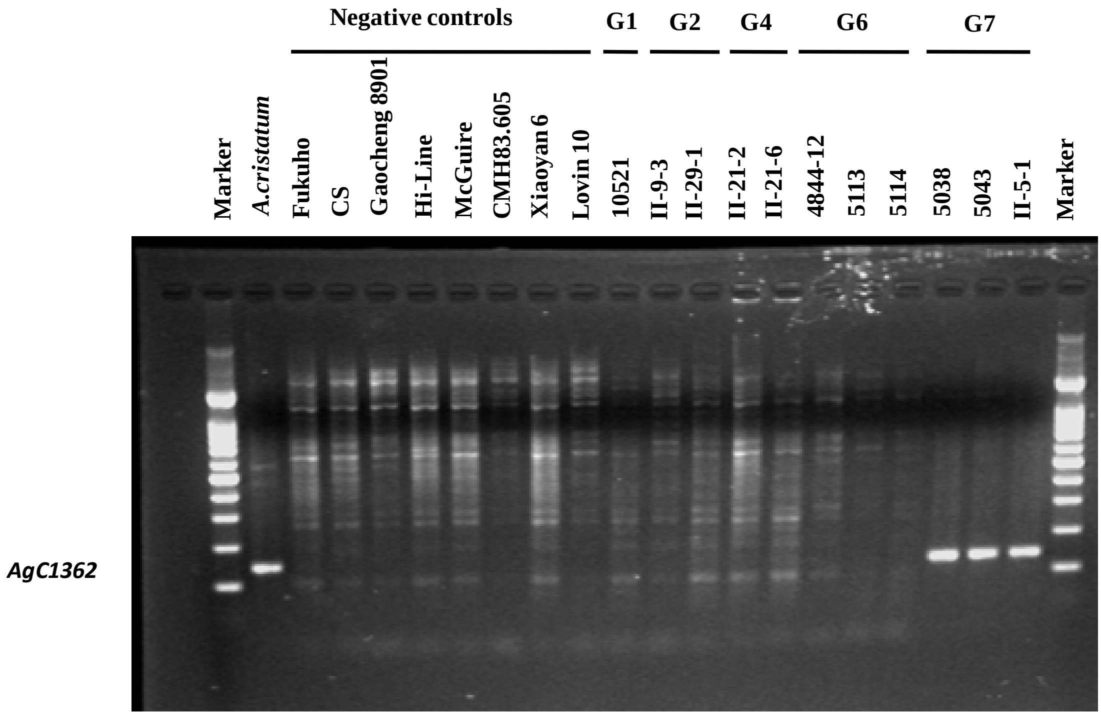

Supplementary Figure 11. The full-length gels of EST-STS markers AgC1362 specific for *A. cristatum* addition lines in wheat genetic backgrounds. The amplification product of EST-STS markers were run on 2% agarose gel. G1, G2, G4, G6, and G7 indicate the homologous groups of alien chromosomes of *A. cristatum*.
